# Predicting Risk of Mortality in Pediatric ICU Based on Ensemble Step-Wise Feature Selection

**DOI:** 10.34133/2021/9365125

**Published:** 2021-05-31

**Authors:** Shenda Hong, Xinlin Hou, Jin Jing, Wendong Ge, Luxia Zhang

**Affiliations:** ^1^National Institute of Health Data Science at Peking University, Beijing, China; ^2^Institute of Medical Technology, Health Science Center of Peking University, Beijing, China; ^3^Neonatology Department of Peking University First Hospital, BeijingChina; ^4^Harvard Medical School, Boston, MA, USA; ^5^Clinical Data Animation Center (CDAC), Massachusetts General Hospital, Boston, MA, USA

## Abstract

*Background*. Prediction of mortality risk in intensive care units (ICU) is an important task. Data-driven methods such as scoring systems, machine learning methods, and deep learning methods have been investigated for a long time. However, few data-driven methods are specially developed for pediatric ICU. In this paper, we aim to amend this gap—build a simple yet effective linear machine learning model from a number of hand-crafted features for mortality prediction in pediatric ICU.*Methods*. We use a recently released publicly available pediatric ICU dataset named pediatric intensive care (PIC) from Children’s Hospital of Zhejiang University School of Medicine in China. Unlike previous sophisticated machine learning methods, we want our method to keep simple that can be easily understood by clinical staffs. Thus, an ensemble step-wise feature ranking and selection method is proposed to select a small subset of effective features from the entire feature set. A logistic regression classifier is built upon selected features for mortality prediction.*Results*. The final predictive linear model with 11 features achieves a 0.7531 ROC-AUC score on the hold-out test set, which is comparable with a logistic regression classifier using all 397 features (0.7610 ROC-AUC score) and is higher than the existing well known pediatric mortality risk scorer PRISM III (0.6895 ROC-AUC score).*Conclusions*. Our method improves feature ranking and selection by utilizing an ensemble method while keeping a simple linear form of the predictive model and therefore achieves better generalizability and performance on mortality prediction in pediatric ICU.

## 1. Introduction

Data-driven methods have been developed for mortality prediction in intensive care units (ICU) for a long time. Traditionally, table-based scoring systems such as Acute Physiology and Chronic Health Evaluation (APACHE) III score [1], Simplified Acute Physiology Score (SAPS) II [2], and Sequential Organ Failure Assessment (SOFA) [3] are more welcomed by clinical staffs because they are easy to calculate and understand. Recently, more sophisticated data-driven methods such as machine learning methods [4, 5], deep learning methods [4, 6-12], and ensemble methods [7, 9, 13] have been proposed for more accurate mortality prediction based on much larger public available ICU datasets such as Medical Information Mart for Intensive Care (MIMIC-III) [14] and eICU Collaborative Research Database (eICU) [15].

However, few data-driven methods are specially developed for pediatric ICU. For general ICU patients, some methods mixed the whole population without age stratification [4, 6, 8], others removed patients who are younger than an age threshold [5, 7, 13]. In fact, child patients in pediatric ICU have quite different physiological conditions compared with other patients. For example, hypernatremia is more severe for adult patients than neonate. So, we cannot bring general ICU models into pediatric ICU directly. Besides, although sophisticated machine learning methods usually get higher prediction accuracy, they are on the shelf because clinical staffs cannot fully understand such black-box models [16]. The initial purpose of mortality prediction is to not play its role at all. In contrast, linear models and tree models are more interpretable and welcomed by clinical staffs—they can easily understand the predictions of simple models from mathematics.

In this paper, we aim to build a simple yet effective linear machine learning model from a number of hand-crafted features for mortality prediction in pediatric ICU. The model is trained based on a recent released pediatric ICU dataset named pediatric intensive care (PIC) database [17]. Unlike previous sophisticated machine learning methods such as random forest (RF) [18] classifier, gradient boosting machine (GBM) [19] classifier, or deep neural networks, we want our method to be kept simple that can be easily understood by clinical staffs. To achieve this, we first extract 397 hand-crafted features including demographics, routine vital signs, input and output, and laboratory values. Then, we propose an ensemble step-wise feature ranking and selection method to rank and select a small subset of effective features from the entire feature set. Finally, we build a logistic regression (LR) classifier upon selected features. The ensemble feature ranking and selection also promote the model generalizability so that it also performs well on the hold-out test set.

As a result, one of the final predictive linear model using 11 features achieves 0.7531 area under the receiver operating characteristic curve (ROC-AUC) score on hold-out test set, which is comparable with the linear model using all 397 features (0.7610 ROC-AUC score), and is higher than existing well known pediatric mortality risk scorer PRISM III (0.6895 ROC-AUC score). Another linear model using the best selected features (59 features) achieves a 0.7885 ROC-AUC score on the hold-out test set, which is the highest among all models. We release details of the models and hope they can be implemented for pediatric ICU mortality prediction in the real world.

## 2. Materials and Methods

### 2.1. Dataset

We use a recently released publicly available pediatric ICU dataset named PIC [17]. It comprises information relating to patients admitted to critical care units at the Children’s Hospital of Zhejiang University School of Medicine in China. Existing data-driven analysis on PIC data include metabolic acidosis of children with acute kidney injury [20], timing/duration of tracheal intubation, and mechanical ventilation on mortality of children [21]. This paper will focus on a different task—mortality prediction. 

**Figure 1:**
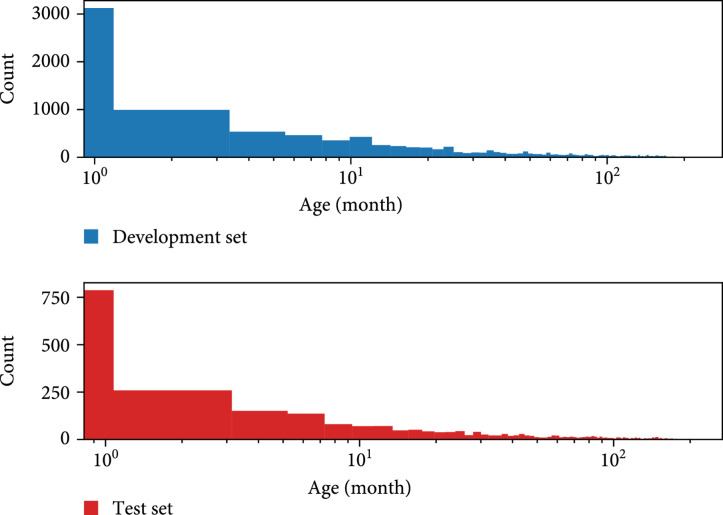
Age distribution of development set and test set (x-axes are log-scaled).

We first exclude 191 patients whose mortality outcomes occurred within the first 24 hours following admission in the experiments. Then, we split the data into 80% development (for model training and validation) set, 20% test set (for evaluation). Mortality outcomes are determined by HOSPITAL_EXPIRE_FLAG in the ADMISSIONS table. Summaries of the patient characteristics of the development set and test set are presented in Table 1. The age distribution of the development set and test set is shown in Figure 1. 

**Table 1:** Patient characteristics of development set and test set.

Demographic characteristics	Development set	Test set
# of subjects	10606	2652
Age in month (mean, std)	30.24, 44.35	28.96, 42.71
Gender		
Male (#, %)	6081, 42.66%	1535, 42.12%
Female (#, %)	4525, 57.44%	1117, 57.88%
In-hospital mortality (#, %)	630, 5.94%	150, 5.66%

### 2.2. Feature Extraction

We extract demographic information as features from patient’s admission information and charted data. Besides, we also extract temporal values from routine vital signs, input and output, laboratory values, and transform them into features by taking min, max, and range values during the first 24 hours after admission. Features with more than 90% missing rates are removed. Consequently, the number of prepared hand-crafted features is 397. 

### 2.3. Ensemble Step-Wise Feature Ranking and Selection

Feature selection can simplify the final model, which makes the predictive model easier understood and accepted by clinical staffs [22]. Feature selection can also promote the generalizability of the model, thus, leads to better performance on beyond development set (training data and validation data). Recent papers tried to promote the quality of feature selection by using ensemble methods [23, 24]. However, it is hard for many feature selection algorithms to control the number of output features, as they usually have other indirectly related hyperparameters. 

**Figure 2:**
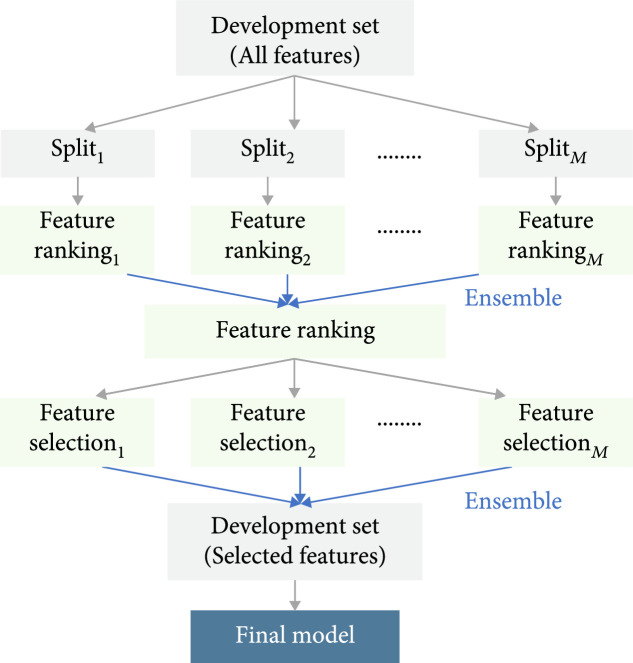
Framework of ensemble step-wise feature ranking and selection.

Here, we propose an ensemble step-wise feature ranking and selection algorithm to control the number of output features in the development set. The framework is shown in Figure 2. The pseudocode is shown in Algorithm 1. 

In the next sections, we will first introduce traditional feature ranking. Then, it leads to our ensemble feature ranking. Finally, we will show step-wise ensemble feature selection based on ranked features and build a machine learning model on selected features as the final mortality predictor.

**Figure 3:**
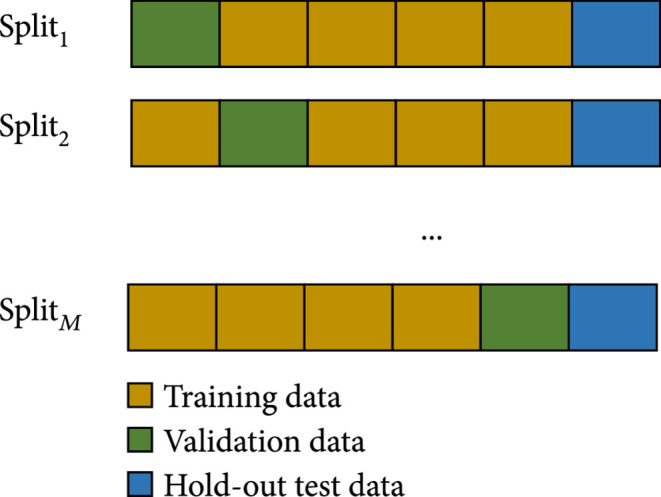
The diagram of training, validation, and test split.

**Algorithm 1: Ensemble step-wise feature ranking and selection.**
1: **Input**: Development set $D=\left\{x,y\right\}$, where $x\in {\mathbb{R}}^{n\times d}$ is the data (n is the number of samples, d is the number of features), $y\in {\left\{0,1\right\}}^{n}$ is corresponding outcome.
2: **Parameters**: Maximum selected features K.
3: **Output**: Mortality predictor F.
4: Split D into M subsets based on cross validation in Figure 3, the ith training set is $D_{i}=\left\{x_{i},y_{i}\right\}$, validation set is $T_{i}=\left\{x_{i},y_{i}\right\}$.
5: **for** i = {1,⋯,M} **do.**
6:  Build feature ranker $H_{i}$ based on $D_{i.}$
7: **end for**
8: Get ensemble feature ranker H from $H_{1},\cdots ,H_{M}$ based on Equation (2).
9: **for** j = {1,⋯,K} **do**
10:  Select top j features based on H, compose development set $D_{j}=\left\{x_{j},y_{j}\right\}$, where $x_{j}\in {\mathbb{R}}^{n\times j}$
11:  Split $D_{j}$ into M subsets based on cross validation in Figure 3, the ith training set is $D_{ji}=\left\{x_{ji},y_{ji}\right\}$, validation set is $T_{ji}=\left\{x_{ji},y_{ji}\right\}$.
12:   **for** i = {1,⋯,M} **do**
13:   Training mortality predictor $F_{ji}$, evaluate on validation set $T_{ji}$
14:   **end for**
15:  Compute average performance of predictors with top i features
16: **end for**
17: Output top i features who has the best performance.

#### 2.3.1. Feature Ranking

We use GBM to rank feature importance. Each feature importance is calculated by the total reduction of the criterion from that feature, which is usually referred to as the average information gain (Gini importance) [19,25].

Formally, denote the feature ranker as H. The input is $x\in {\mathbb{R}}^{n\times d}$, where n is the number of samples, and d is the number of features. Then, the output is $s\in {\mathbb{R}}^{d}$, where $s\left[i\right]$ represents the ith feature importance score.

(1)s=Hx.

#### 2.3.2. Ensemble Feature Ranking

The ensemble method [26] can improve the performance of a single predictor, taking bagging [27] as an example. Formally, denote the ith predictor as $P_{i}$, the input is x and output is $y_{i}$. Then, we can get $y_{i}=P_{i}\left(x\right)$. Suppose we have M predictors, the ensemble output y based on bagging is calculated as $y=1/M\sum_{i=1}^{M}\left(y_{i}\right)$.

Given development set $D=\left\{x,y\right\}$, where $x\in {\mathbb{R}}^{n\times d}$, we split the entire dataset into M pieces of subsets, where 1 fold is validation set, and others are training set. The diagram is shown in Figure 3. Thus, we have M splits of training/validation set from the development set. Then, we can build M feature ranker based on them.

Here, we introduce bagging into feature ranking. Now, we have M feature ranker $H_{i}$ where $i=\left\{1,2,\cdots ,M\right\}$, each feature ranker takes $x_{i}$, a subset of x, as input and output feature importance scores $s_{i}$. In implementation, we will split the entire development set into pieces by cross-validation, then compose a subset $x_{i}$ from them. Then, the ensemble feature score s is

(2)s=1M∑i=1Msi=1M∑i=1MHixi.

Finally, we will use s to rank features, which is more reliable than each single $s_{i}$ based on ensemble theory.

#### 2.3.3. Step-Wise Ensemble Feature Selection

Given s, we can get the importance rank of features. Now, we will determine the number of select features based on it. This is done by step-wise ensemble feature selection. Intuitively, “step-wise” means we iteratively add one next feature to the current selected feature set; “ensemble” means we selected features by ensembling M predictors based on split shown in Figure 3.

In detail, at each iteration, we first select top j features based on feature ranker H to compose development set $D_{j}=\left\{x_{j},y_{j}\right\}$, where $x_{j}\in {\mathbb{R}}^{n\times j}$. Then, we split $D_{j}$ into M subsets. The ith training set is $D_{ji}=\left\{x_{ji},y_{ji}\right\}$, validation set is $T_{ji}=\left\{x_{ji},y_{ji}\right\}$. Next, we train mortality predictor $F_{ji}$ based on $D_{ji}$ and evaluate on validation set $T_{ji}$. Finally, we compute the average performance of predictors who have top i features and output top i features who has the best performance.

### 2.4. Mortality Risk Predicting Model

To keep the final model simple and interpretable, we choose F to be logistic regression (LR) classifier. Hence, the predictors in step-wise feature selection are also LR classifier. To overcome the class imbalance problem, we use the inverse proportion of class frequencies to adjust sample-wise weights of the objective function. 

Equation (3) gives the final model, where x is input features. Sigmoid is an activation function $\text{sigmoid}\left(x\right)=1/1+e^{-x}$. The details of each model can be found at https://github.com/hsd1503/PIC_mortality.

(3)p=sigmoidwTx+b.

### 2.5. Evaluations

We use the following measurements: receiver operating characteristic (ROC) curves, area under the ROC curves (ROC-AUC), precision-recall (PR) curves, and area under the PR curves (PR-AUC). For the development set, we further split it into 10 folds, where 1 fold is the validation set, and others are the training set. We impute missing values with the population median. No scale of original features to keep interpretability.

## 3. Results

**Figure 4:**
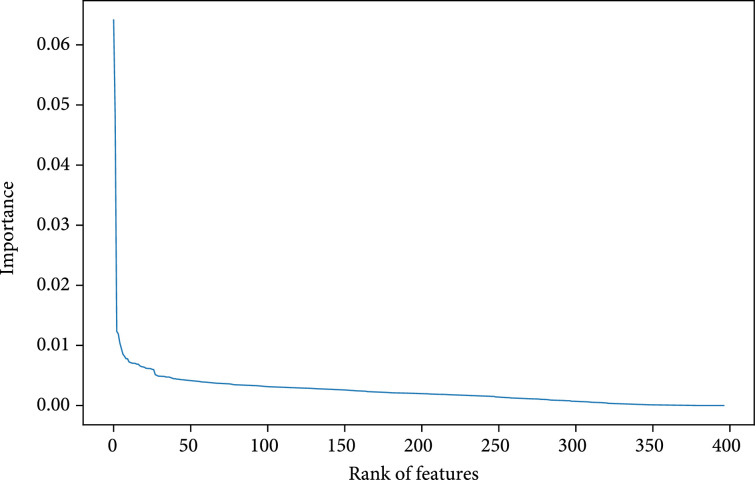
Importance score of features in descent order.

Figure 4 shows the importance score of all 397 features in descent order. The x-axis is features, and the y-axis is feature importance score. The curve decreases rapidly from over 0.06 to around 0.005, then approaching 0 at around 350 features.

**Figure 5:**
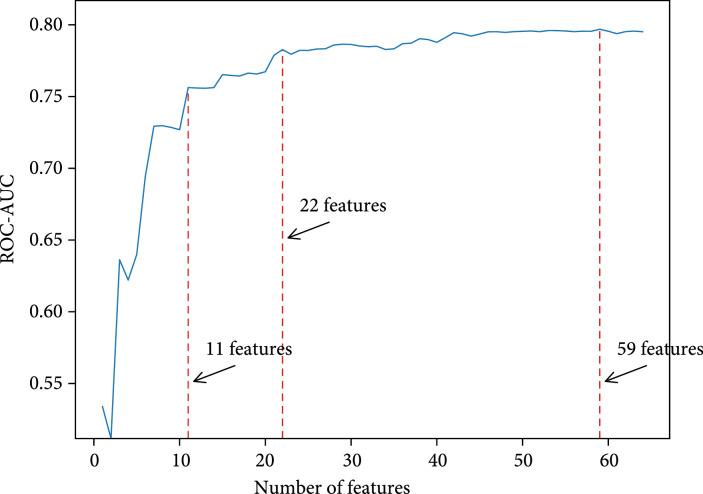
Performance of predictors in step-wise ensemble feature selection process.

Figure 5 shows the performance of predictors in the step-wise ensemble feature selection process. The x-axis is the number of features used in predictors, which is LR in this case. The y-axis is the average ROC-AUC score of predictors on the 10-fold validation set. We can see that the performance increases rapidly in the beginning and reaches platforms at 11 features and 22 features. We select minimal features by using the top 11 features, medium features by using the top 22 features, and best features by using the top 59 features.

Figure 6 shows ROC curves, ROC-AUC scores, PR curves, and PR-AUC scores of different models evaluated on the same hold-out test set. The compared methods are LR with minimal features, LR with medium features, LR with best features, LR with all features, and a well-known pediatric mortality risk scorer PRISM III [28]. We can see that LR best achieves the highest 0.7885 ROC-AUC score. LR minimal is comparable with LR all but it uses much less features. PRISM III achieves a 0.6895 ROC-AUC score.

Figure 6(a) ROC curves, average, and standard deviation ROC-AUC scores of different models. (b) PR curves, average scores, and standard deviation PR-AUC scores of different models.

**(a):**
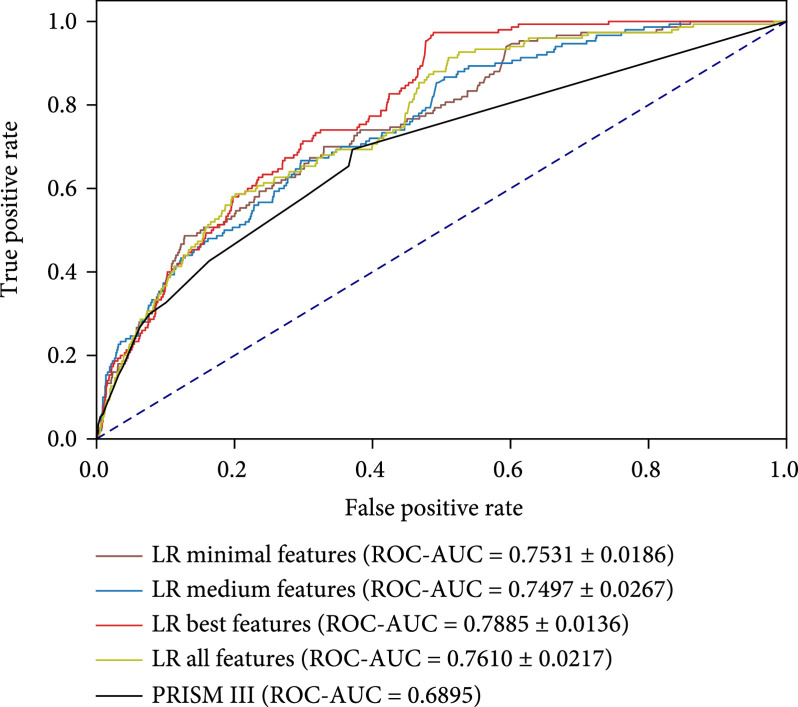

**(b):**
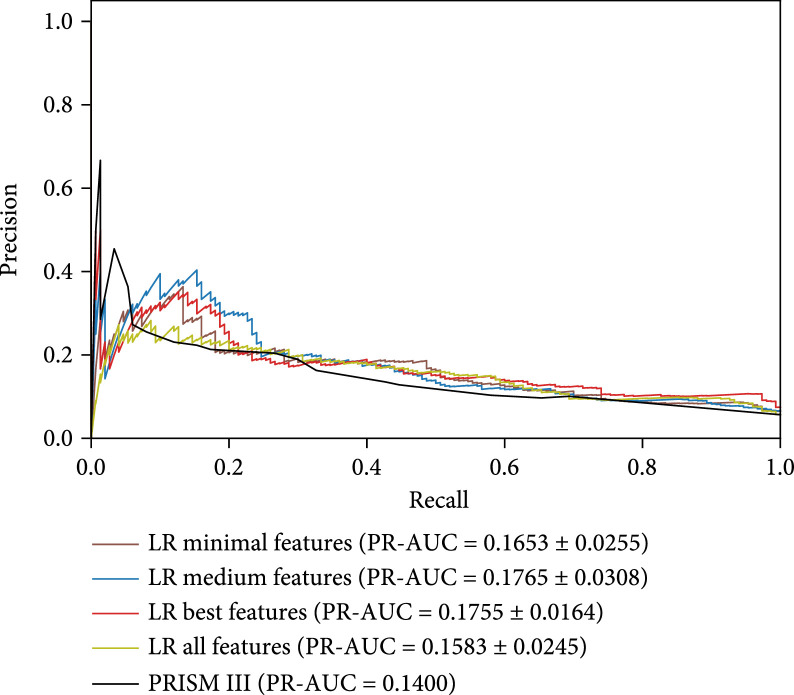

## 4. Discussions

We extract 397 features from the PIC dataset in the beginning, but only a small fraction of features are truly useful to predict mortality risk, while most features do not contribute too much. In Figure 4, a large majority of features are recognized as much less important than a few top features. This result is further verified by step-wise feature selection in Figure 5. The model achieves the highest performance when using the top 59 features, then dropping a little when adding more features. If all features are considered in the LR model, the ROC-AUC score is 0.7610 on the test set, which is only comparable with LR minimal.

Although some machine learning methods such as RF have the ability of feature selection when building the model, so that it can keep a good performance when modeling 397 features simultaneously. However, the final model is too sophisticated to be understood by humans, which hampers it to be implemented in the real world scenario. Besides, the high complexity of the model might also sacrifice the model generalizability.

Our ensemble feature selection has several advantages. First, feature ranking and feature selection are two steps to avoid overfitting, while such phenomena might happens in RF or (Least Absolute Shrinkage and Selection Operator) LASSO, as they simultaneously ranking, selecting, and build model. Second, use an ensemble of multiple folds further improving the generalizability of trained models. Third, the step-wise strategy can be used to control the number of features if special requirements are needed.

Our study has limitations. First, the dataset was collected from a single-center pediatric-specific hospital in China. Although it is a tertiary hospital and covers a broad area of East China, the population might not fully present Chinese children’s demographics. Second, many features have high missing values ratios due to relatively low quality in the ICU dataset. In this paper, we impute missing values with population median. It might lead to inevitable biases in the model. Third, we did not perform clinical experiments in this paper. It will become our main direction for future work.

## 5. Conclusion

In this paper, we present the first work on the PIC dataset that build an interpretable machine learning model for mortality prediction in pediatric ICU. We propose an ensemble step-wise feature selection method to select a small subset of effective features from 397 features and then build a simple linear model for prediction.

In the future, we consider several ways for deployment in real-world applications. First, we will integrate this model into the existing Health Information System (HIS) and let the computer calculate. Second, we plan to simplify the LR model to be a table-based scoring system, so that clinical staffs can calculate it by hand. Moreover, we also plan to adjust weights and reduce features for better-customized deployment.
